# Transcriptome-Wide Association Study Reveals Potentially Candidate Genes Responsible for Milk Production Traits in Buffalo

**DOI:** 10.3390/ijms25052626

**Published:** 2024-02-23

**Authors:** Kelong Wei, Ying Lu, Xiaoya Ma, Anqian Duan, Xingrong Lu, Hamdy Abdel-Shafy, Tingxian Deng

**Affiliations:** 1 Guangxi Provincial Key Laboratory of Buffalo Genetics, Breeding and Reproduction Technology, Buffalo Research Institute, Chinese Academy of Agricultural Sciences, Nanning 530001, China; wei151281938@163.com (K.W.); 18777152038@163.com (Y.L.); maxiaoya8899@163.com (X.M.); duanaq321@163.com (A.D.); luxingrong074@163.com (X.L.); 2 Department of Animal Production, Faculty of Agriculture, Cairo University, Giza 12613, Egypt; hamdyabdelshafy@agr.cu.edu.eg

**Keywords:** buffalo, causal genes, milk production traits, GWAS, TWAS

## Abstract

Identifying key causal genes is critical for unraveling the genetic basis of complex economic traits, yet it remains a formidable challenge. The advent of large-scale sequencing data and computational algorithms, such as transcriptome-wide association studies (TWASs), offers a promising avenue for identifying potential causal genes. In this study, we harnessed the power of TWAS to identify genes potentially responsible for milk production traits, including daily milk yield (MY), fat percentage (FP), and protein percentage (PP), within a cohort of 100 buffaloes. Our approach began by generating the genotype and expression profiles for these 100 buffaloes through whole-genome resequencing and RNA sequencing, respectively. Through comprehensive genome-wide association studies (GWAS), we pinpointed a total of seven and four single nucleotide polymorphisms (SNPs) significantly associated with MY and FP traits, respectively. By using TWAS, we identified 55, 71, and 101 genes as significant signals for MY, FP, and PP traits, respectively. To delve deeper, we conducted protein–protein interaction (PPI) analysis, revealing the categorization of these genes into distinct PPI networks. Interestingly, several TWAS-identified genes within the PPI network played a vital role in milk performance. These findings open new avenues for identifying potentially causal genes underlying important traits, thereby offering invaluable insights for genomics and breeding in buffalo populations.

## 1. Introduction

Water buffalo (*Bubalus bubalis*) represents a cornerstone in domesticated livestock, playing a pivotal role in various global regions due to its economic significance and cultural value. Valued for milk, meat, and draft power, water buffalo constitute a crucial source of income for smallholder farmers. The growing global demand for buffalo milk, particularly in Europe for premium cheeses and other dairy products, underscores its economic importance. To sustain and further improve the buffalo’s milk production performance, understanding the genetic basis of these traits is of paramount importance. Currently, this remains poorly understood, presenting a significant knowledge gap in buffalo genetics and breeding research. With the rapid advancement of genome-wide association studies (GWASs), numerous genetic variants related to important economic traits in buffalo populations have been continuously identified. These traits encompass milk production traits [1,2,3], reproductive traits [2,4,5], and growth traits [6]. While GWAS has provided valuable insights, it is constrained by its limitations in uncovering causal variants [7,8,9]. As a result, the identification of causal loci remains a challenge, and many discoveries have remained at the suggestive level, lacking validation across different studies.

Given the limitations of GWAS in identifying causal genetic variants, omics-based association analyses have emerged, offering greater statistical power in the quest to detect causal genes or loci. Over the last decade, transcriptome-wide association studies (TWAS) have risen to prominence as a potent approach for identifying causal variants that influence gene expression and complex traits [10]. By integrating GWAS summary statistics and gene expression data from reference panels, TWAS can effectively prioritize potentially causal genes associated with traits [11]. Additionally, by using the TWAS approach, it is possible to identify candidate genes that are not only linked to disease risk but also under the regulation of genetic variants. For example, in a study by Al-Barghouthi et al. [12], *PPP6R3* was identified as a potential causal gene for human bone mineral density. Similarly, Cai et al. [13] discovered several TWAS genes as candidates for important economic traits in Simmental beef cattle, including *NADSYN1*, *NDUFS3*, *LTF*, and *KIFC2* in the liver, *GRAMD1C*, *TMTC2*, and *ZNF613* in backfat, as well as *TIGAR*, *NDUFS3*, and *L3HYPDH* in muscle. Chhotaray et al. [14] reported some causal loci related to milk production and composition traits in Murrah buffaloes. These findings emphasize the potential of TWAS in uncovering genes underlying complex traits, making it crucial for unraveling the genetic basis of milk production traits in buffalo populations and ultimately addressing the limitations encountered by previous GWAS.

TWAS is a gene-prioritization method designed to detect trait-related genes regulated by GWAS signals [15]. The standard TWAS contains two stages. Stage I involves training multivariable regression models on reference data, treating gene expression as an outcome and SNP genotypes (typically *cis*-SNPs near the test gene) as predictors to determine genetically regulated expression (GReX). Stage II imputes GReX in an independent GWAS of a complex trait using the trained expression quantitative trait loci (eQTL) effect sizes. For TWAS stage I, a variety of training tools have been developed, including PrediXcan [16], FUSION [17], and TIGAR-V2 [18]. PrediXcan employs the general linear regression model with Elastic Net penalty, while FUSION incorporates Elastic-Net, LASSO, linear mixed modeling, SuSiE (sum of single effects), and BSLMM (Bayesian sparse linear mixed model) to estimate the effect size. In contrast, TIGAR-V2, by employing a nonparametric Bayesian Dirichlet process regression (DPR) model, offers a more comprehensive approach that captures a broader range of genetic effects. The DPR introduces an unknown distribution on the variance parameter of SNP effect size (*β*) and estimates *β* based on the input data rather than relying on parametric priors. Remarkably, Mai et al. [15] demonstrated that the non-parametric DPR model significantly enhances the power of TWAS, outperforming PrediXcan.

In light of the strengths offered by TWAS and the innovative approaches it encompasses, the objective of this study is to harness TWAS for the identification of candidate causal genes associated with milk production traits in water buffalo. This endeavor seeks to address the limitations of previous GWAS and, in turn, to bridge the current knowledge gap within the field of buffalo genetics and breeding research. The ultimate aim is to make substantial contributions to the sustainable enhancement of buffalo milk production performance. In pursuit of our objective, we conducted a comprehensive TWAS using a dataset of 100 buffaloes. This involved whole-genome resequencing and RNA sequencing for the 100 buffalo samples, followed by GWAS for milk production traits. Subsequently, we estimated the *cis*-eQTL effect through nonparametric Bayesian DPR and performed TWAS using summary-level GWAS data to identify the causal genes.

## 2. Results

### 2.1. Genomic Profiling and eQTL Weight Analysis

**Figure 1:**
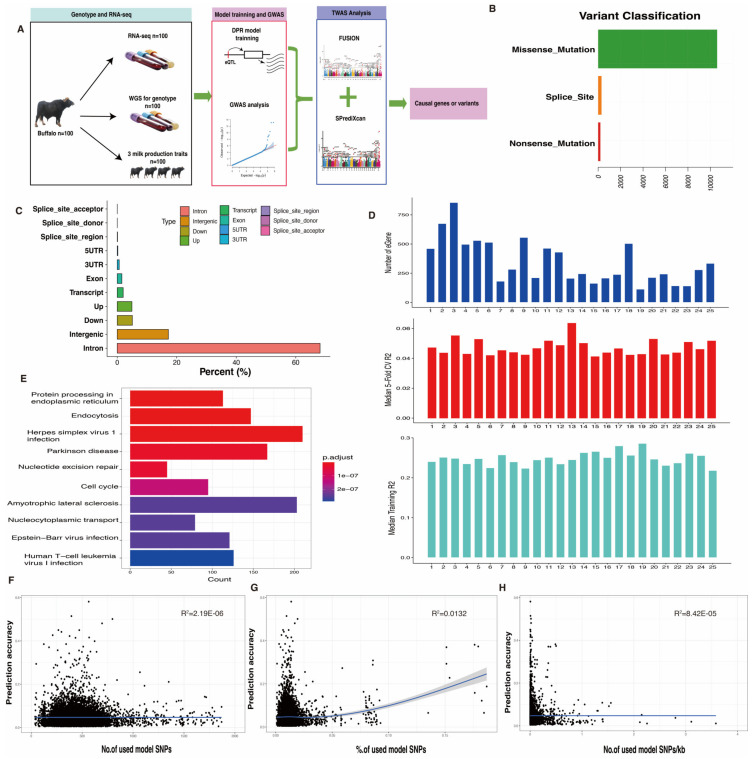
Study design and eQTL characterization in buffalo samples. (**A**) Design overview of transcriptome-wide association study for milk production traits in buffalo. (**B**,**C**) SNP distribution types in buffalo whole-genome sequencing data. (**D**) eGene distribution and prediction accuracy across buffalo chromosomes. (**E**) Top 10 KEGG functional enrichment analysis for eQTL-regulated genes. (**F**–**H**) Scatter plots for the relationship between prediction accuracy model properties.

In this study, we generated an impressive 9.35 billion clean reads from WGS and 6.69 billion clean reads from RNA-seq, using a dataset of 100 whole blood samples. Detailed information on WGS and RNA-seq data can be found in Table S1. Within the WGS dataset, a total of 679,118 SNPs successfully passed our quality control criteria. Among these, 10,281 SNPs, accounting for 56.94% of the total, were classified as missense mutation variants (Figure 1B). The majority of SNPs in this dataset fell into the category of intron or intergenic variants (Figure 1C). For the RNA-seq dataset, 14,315 genes, equivalent to 38.55% of the total, met the expression threshold of TPM ≥ 0.1 in at least 20% of the samples. These filtered SNPs and genes were subsequently used for the prediction of tissue-specific gene expression models.

Blood-specific gene expression prediction models for buffalo were trained using the nonparametric Bayesian DPR method, implemented in the TIGAR v2 tool. From the filtered expression data, a total of 8687 “significant” gene expression prediction models with a 5-fold cross-validation (CV) R2 (r-square) > 0.005 were successfully developed using the DPR model. The number of eGenes in the prediction models varied, with the highest at 855 and the lowest at 113 for different chromosomes (Figure 1D, top), resembling the gene distribution in the buffalo genome. In addition, the median 5-fold CV R2 exceeded 0.04 per chromosome, as shown in the central panel of Figure 1D, while the median training R2 of genome-wide genes surpassed 0.2, as depicted in the lower panel of Figure 1D. To gain insight into the potential biological functions of the *cis*-eQTL target genes, a gene functional enrichment analysis was conducted. It was observed that eGenes were significantly enriched (*p*-value < 0.05) in common KEGG pathways, including protein processing in the endoplasmic reticulum, endocytosis, and herpes simplex virus 1 infection (Figure 1E).

Furthermore, we explored whether prediction accuracy was influenced by specific model properties, such as the inclusion of more variants in the input genotypic data for expression prediction. To address this, we investigated the relationships between prediction accuracy and three model properties: (i) the number of SNPs used for model prediction (Figure 1F); (ii) the percentage of SNPs used for model prediction (Figure 1G); and (iii) the number of SNPs used, adjusted for gene length (Figure 1H). While incorporating more variants to predict gene expression levels led to a slight improvement in prediction accuracy, the relationships were relatively weak, rendering these model properties ineffective for assessing or improving TWAS predictions.

### 2.2. Identification of Causal Genes for Milk Yield

**Figure 2:**
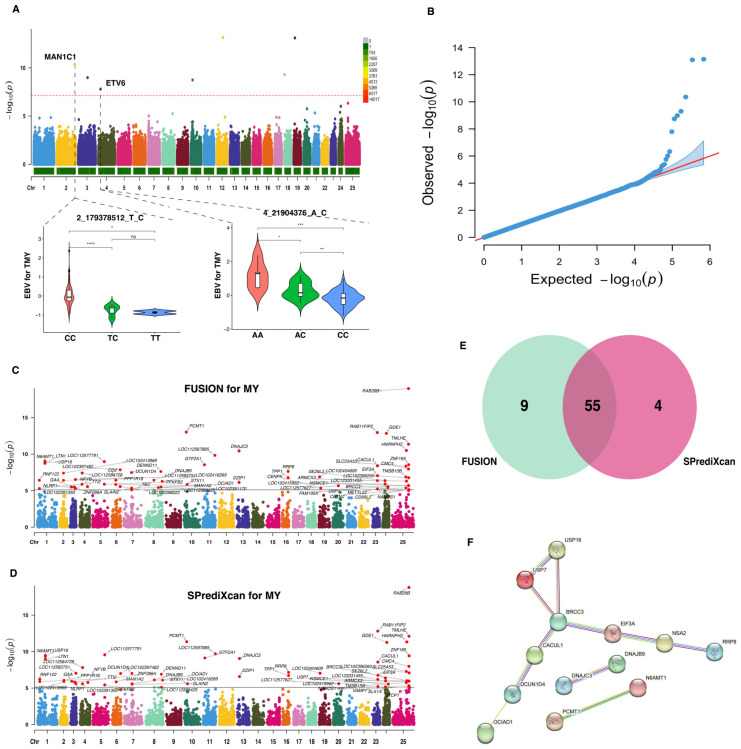
Genome-wide and transcriptome-wide association studies for milk yield in buffalo. (**A**) Manhattan plot based on GWAS for MY trait. The horizontal red dashed line represents the genome-wide significance threshold at −log_10_(7.36 × 10^−8^). Below this figure, a distribution of genotype frequencies is displayed for the most important SNPs identified in the GWAS. (**B**) QQ-plot for *p*-values based on GWAS for MY trait. (**C**) Manhattan plot based on TWAS for MY trait using FUSION analysis. The horizontal gray line indicates the genome-wide significance threshold at −log_10_(7.83 × 10^−6^). (**D**) Manhattan plot based on TWAS for MY trait using SPrediXcan analysis. The horizontal gray line indicates the genome-wide significance threshold at −log_10_(7.83 × 10^−6^). (**E**) Venn diagram showing the overlapped genes. (**F**) Protein–protein interaction network analysis of the overlapped genes. * indicates *p*-value < 0.05, ** indicates *p*-value < 0.01, *** indicates *p*-value < 0.001, **** indicates *p*-value < 0.0001.

Prior to TWAS analysis, we conducted a GWAS for the MY trait in buffalo that revealed a total of 53 SNPs with nominal association (*p*-value < 1 × 10^−4^). Seven of these SNPs (Figure 2A; Table 1) were significant and passed the Bonferroni threshold level (*p*-value < 7.36 × 10^−8^). The reliability of the GWAS analysis for the MY trait is shown in Figure 2B, which includes a QQ plot providing a visual assessment of the concordance between the expected distribution of *p*-values under the null hypothesis and the observed distribution from the GWAS. In addition, it was observed that two SNPs, specifically 2_179378512_T_C and 4_21904376_A_C, were located within the genetic regions of the *MAN1C1* and *ETV6* genes, respectively. Notably, the genotypes CC and AA were identified as the dominant allele types for the SNPs 2_179378512_T_C and 4_21904376_A_C, respectively.

Utilizing the GWAS summary statistics for MY trait and Bayesian blood *cis*-eQTL weights, we detected 64 and 59 significant TWAS genes (*p*-values < 7.83 × 10^−6^) using FUSION (Figure 2C) and SPrediXcan (Figure 2D), respectively. Among these significant genes, 55 were common to both methods (Figure 2E), which were defined as causal genes related to the MY trait. Further details regarding these candidate genes are listed in Table S2. The PPI analysis revealed that these genes could be grouped into four distinct networks, with the *BRCC3* genes having the most nodes (Figure 2F).

### 2.3. Identification of Causal Genes for Fat Percentage

**Figure 3:**
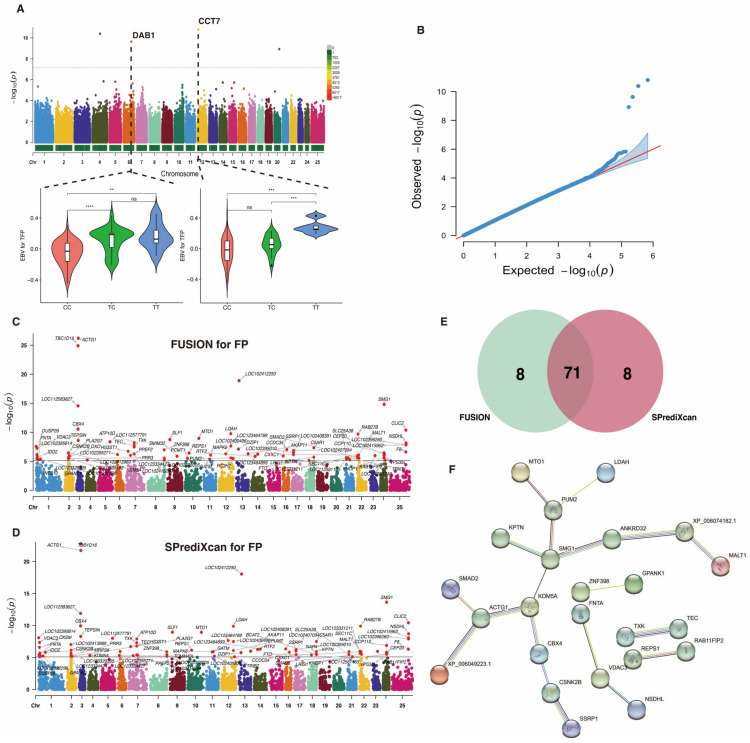
Genome-wide and transcriptome-wide association studies for fat percentage in buffalo. (**A**) Manhattan plot based on GWAS for the FP trait. The horizontal red dashed line represents the genome-wide significance threshold at −log_10_(7.36 × 10^−8^). Below this figure, a distribution of genotype frequencies is displayed for the most important SNPs identified in the GWAS. (**B**) QQ-plot for *p*-values based on GWAS for the FP trait. (**C**) Manhattan plot based on TWAS for the FP trait using FUSION analysis. The horizontal gray line indicates the genome-wide significance threshold at −log_10_(7.86 × 10^−6^). (**D**) Manhattan plot based on TWAS for the FP trait using SPrediXcan analysis. The horizontal gray line indicates the genome-wide significance threshold at −log_10_(7.86 × 10^−6^). (**E**) Venn diagram showing the overlapped genes. (**F**) Protein–protein interaction network analysis of the overlapped genes. ** indicates *p*-value < 0.01, *** indicates *p*-value < 0.001, **** indicates *p*-value < 0.0001.

To uncover potential candidate genes associated with the FP trait, we conduct a TWAS based on the gene-regulated expression of this trait. Prior to TWAS analysis, our GWAS detected a total of 83 SNPs nominally related to the FP trait (*p*-value < 1 × 10^−4^), with 4 of them being significantly associated at *p*-value < 7.36 × 10^−8^ (Figure 3A; Table 2). The reliability of the GWAS for the FP trait is depicted in Figure 3B, which includes a QQ plot providing a visual assessment of the concordance between the expected distribution of *p*-values under the null hypothesis and the observed distribution from the GWAS. Two SNPs (6_88599507_T_C and 12_11038738_T_C) were found to be located in the genetic regions of *DAB1* and *CCT7*, respectively. Notably, the genotype TT was the dominant allele type in 6_88599507_T_C and 12_11038738_T_C, respectively.

Leveraging the GWAS summary statistics of the FP trait and Bayesian blood *cis*-eQTL weights, we detected 79 significant TWAS genes (*p*-values < 7.86 × 10^−6^) using both FUSION (Figure 3C) and SPrediXcan (Figure 3D). Among these, 71 candidate genes overlapped between both methods (Figure 3E), and these were designated as causal genes responsible for the variation in the FP trait. Additional information for these candidate genes can be found in Table S3. An analysis of PPI revealed that these genes clustered into five networks, with the *SMG1* genes having the most nodes (Figure 3F).

### 2.4. Identification of Causal Genes for Protein Percentage

**Figure 4:**
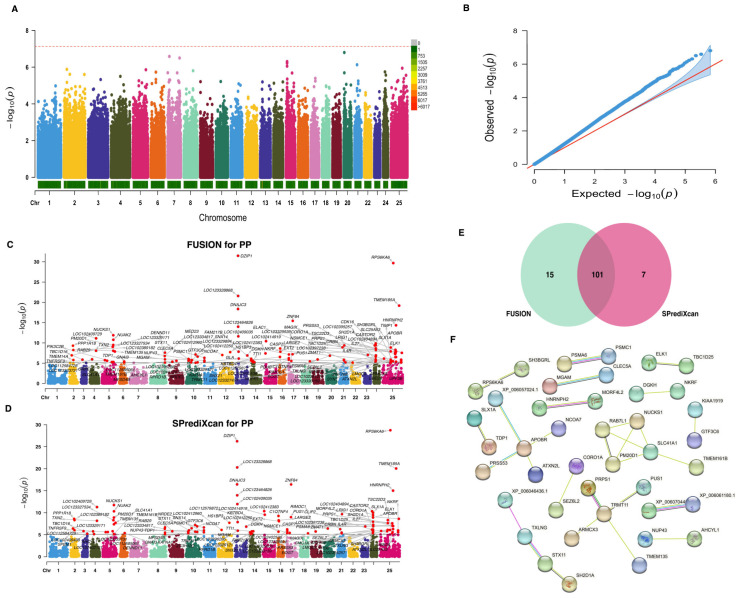
Genome-wide and transcriptome-wide association studies for protein percentage in buffalo. (**A**) Manhattan plot based on GWAS for the PP trait. The horizontal red dashed line represents the genome-wide significance threshold at −log_10_(7.36 × 10^−8^). (**B**) QQ-plot for *p*-values based on GWAS for the PP trait. (**C**) Manhattan plot based on TWAS for the PP trait using FUSION analysis. The horizontal gray line indicates the genome-wide significance threshold at −log_10_(8.03 × 10^−6^). (**D**) Manhattan plot based on TWAS for the PP trait using SPrediXcan analysis. The horizontal gray line indicates the genome-wide significance threshold at −log_10_(8.03 × 10^−6^). (**E**) Venn diagram showing the overlapped genes. (**F**) Protein–protein interaction network analysis of the overlapped genes.

In our quest to identify genes associated with the PP trait, we conducted a TWAS based on gene-regulated expression. Traditional GWAS for this trait uncovered a total of 398 nominally related SNPs (*p*-value < 1 × 10^−4^). However, when considering Bonferroni correction, no significant signals were detected (Figure 4A). Although no significant signals were observed, the QQ plot in Figure 4B reveals a small deviation in the observed *p*-values from the expected distribution under the null hypothesis. Using the GWAS summary statistics for the PP trait and Bayesian blood cis-eQTL weights, we determined 116 and 108 significant TWAS genes (*p*-values < 8.03 × 10^−6^) for this trait using both FUSION (Figure 4C) and SPrediXcan (Figure 4D) approaches, respectively. A total of 101 genes were shared between both methods (Figure 3E), and these genes are considered causal genes associated with the PP trait. For in-depth information on these candidate genes, please refer to Table S4. A PPI analysis revealed that these genes are organized into 15 different networks, with the *SLC41A1* gene exhibiting the highest degree of interconnected nodes (Figure 4F).

## 3. Discussion

While GWASs in buffalo have been extensively used in recent years, the translation of their findings into practical applications within animal breeding remains limited. This limitation is primarily attributed to factors such as small sample sizes, the complexity of the traits under examination, and the scarcity of high-quality records [1,2,3,19,20]. Furthermore, the challenges of extensive linkage disequilibrium and the elusive “missing heritability” phenomenon further hinder the precise identification of causal genetic variants [21,22]. As a result, many previous discoveries in buffalo GWASs have remained at the suggestive level, while few significant loci were detected and did not overlap among different studies, leaving them awaiting validation.

In the current study, we encountered similar circumstances with the identification of significant GWAS signals. Specifically, we discovered seven SNPs significantly associated with MY and four SNPs related to FP. The SNPs linked to MY were located proximate to four protein-coding genes (*MAN1C1*, *ETV6*, *SASH1*, and *VPS54*) and three ncRNA genes (*LOC123332809*, *LOC123330224*, and *LOC112580602*). It is noteworthy that only two SNPs (2_179378512_T_C and 4_21904376_A_C) were positioned within the genetic region of the *MAN1C1* and *ETV6* genes, respectively. Existing evidence supports the role of *MAN1C1* in lactation persistency in Canadian Holstein cattle [23], while *ETV6* is considered a candidate gene affecting fat yield in North American Holstein cattle [4]. Moreover, we found that only three SNPs associated with FP were mapped to protein-coding genes, including *SLC38A1*, *DAB1*, and *CCT7*. Among these, *SLC38A1* has been previously reported for milk protein synthesis [24], and *DAB1* was reported to be associated with mammary gland morphogenesis [25] and fatty acid intake [26], while there is no supporting evidence to establish a connection between *CCT7* and milk fat percentage.

In the present study, we utilized TWAS technology to identify causal genes related to milk production traits within a buffalo population consisting of 100 individuals. We conducted extensive analyses to estimate the effect size of *cis*-eQTL and investigated the factors affecting *cis*-eQTL weights. In this regard, we successfully identified 55, 71, and 101 genes exhibiting significant associations with MY, FP, and PP traits, respectively. Growing evidence suggests that expression quantitative trait loci (eQTLs) are more likely to be found in SNPs linked to complex traits [27]. Incorporating eQTL information, such as eQTL weights, into GWAS has the potential to enhance its power. Estimating eQTL weights can be approached through different modeling techniques, including the Elastic Net model [28], the Bayesian sparse linear mixed model [29], Dirichlet process regression [30], the linear mixed model [31], and Bayesian variable selection regression [32]. In our study, we employed a non-parametric Bayesian DPR strategy to train a gene expression imputation model using 100 buffalo blood samples and estimate *cis*-eQTL weights. Impressively, the DPR model effectively trained 8687 genes, achieving a 5-fold CV R2 >0.005. Additionally, we observed that the median 5-fold CV R2 per chromosome exceeded 0.04, with the training R2 value surpassing 0.2. Both values exceeded those reported in human blood studies conducted by Parrish et al. [18], suggesting the suitability of these *cis*-eQTL weight estimates for further analyses. Furthermore, we explored the potential impact of specific model properties on prediction accuracy. Interestingly, we found that prediction accuracy exhibited a weak relationship with model properties, including the number and percentage of model variants used for prediction, as well as the number of model variants adjusted for gene length. This observation implies that these model properties may not be utilized to improve the prediction performance of TWAS.

It is well established that eQTL analyses, including TWAS, play a pivotal role in interpreting GWAS results and improving the power of identifying GWAS signals [33]. This approach holds immense promise for unraveling functional sequence variations and understanding the fundamental mechanisms of gene regulation [34]. Several genes identified in our TWAS analysis have robust support from prior functional studies and have been linked to known loci associated with milk production traits. For example, *CCDC34* has been proposed as a candidate gene affecting fat percentage in Indian buffaloes [4]. *FTO* has been suggested as a functional signature for fat percentage [35]. Additionally, three genes (*TTI2*, *RNF122*, and *NLRP1*) have been associated with milk yield in buffaloes [8]. A recent study by Wen et al. [36] has demonstrated that copy number variations of the USP16 gene play an important role in milk production traits in Chinese Holstein cattle, underscoring its potential as a molecular marker for assisted selection. Moreover, *NUCKS1* has emerged as a novel regulator of milk synthesis, including milk protein, milk fat, and lactose synthesis [37]. Lastly, both *SLC25A53* and *SLC41A1*, members of the solute carrier family, have been associated with mammary protein synthesis [38].

The identification of causal genes contributing to phenotypic variation significantly advances our understanding of buffalo biology and holds substantial potential for improving buffalo breeding and productivity. To pinpoint the most promising TWAS genes, we further refined our analysis through PPI network analysis. In this regard, a total of 9, 15, and 17 TWAS genes associated with MY, FP, and PP traits, respectively, were classified into large networks (degree nodes > 4). The majority of these genes were found to be directly or indirectly linked to the biological functions associated with mammary gland development and lactation. For example, *USP16* has been identified as a modulator of the Wnt pathway in mammary epithelia [39], thereby influencing mammary gland development and lactation [40]. In Chinese Holstein cattle, Wen et al. [36] found that the copy number variation of the *USP16* gene was associated with milk traits. *BRCC3* plays a crucial role in the modulation of cell survival and proliferation [41]. *EIF3A* is vital for stimulating protein synthesis [42,43]. The silencing of *CSNK2β* significantly inhibits cell growth and induces apoptosis [44], suggesting its critical role in modulating the proliferation and apoptosis of mammary epithelial cells. *SMAD2* is a member of the SMAD protein family, a key intermediary in transforming growth factor beta (TGF-β) signaling [45], which plays a vital role in mammary gland development [46]. Notably, evidence showed that *SMAD2* was associated with goat growth traits [47], sheep litter size [48], and buffalo milk yield traits [49]. In addition, *SMG1* belongs to the phosphatidylinositol 3-kinase-related kinase (PIKK) protein family, triggering the activation of AMPK, which aids in the regulation of milk production and mammary gland biology [50]. These findings strongly indicate that these TWAS genes are correlated with milk performance in buffaloes.

In the present study, a relatively small sample size serves as a limitation, which can be addressed by including larger cohorts in future research. Next, while genetically predicted models were assessed in blood samples, it remains essential to validate these models in biologically relevant tissues such as mammary glands and mammary epithelial cells for more accurate results. Finally, it is worth noting that TWAS may not capture all genes, especially those with SNPs that influence milk production traits independently of cis expression, which may be overlooked. Addressing these limitations paves the way for future investigations seeking a more comprehensive understanding of the genetic basis of buffalo milk production traits in dairy animals.

## 4. Materials and Methods

### 4.1. Animals and Phenotype

A total of 100 buffaloes with complete records were used. These buffaloes were crossbred with the Murrah or Nili-Ravi buffalo and Chinese native buffalo. These animals were sourced from the Guangxi Buffalo Research Institute in Guangxi, China. Over a five-year period (2017–2022), a dataset comprising 2084 test-day records for these buffaloes was collected. All records, including daily milk yield, fat percentage, and protein percentage, were collected and measured by the Hubei Dairy Cattle Performance Measurement Center (Wuhan, China). A minimum of five test-day records within each parity, limited to the first three lactations, was mandated to ensure a comprehensive representation of the phenotypes. Prior to conducting further analyses, it was crucial to adjust for non-genetic factors and obtain a single value for each animal, accurately representing each animal’s phenotype. In this context, all available factors were initially tested for significant effects using a linear model with the ‘lm’ function in R. Only significant factors were retained for use in the subsequent model. In the final model, the estimated breeding values (EBVs) for each individual were computed using a random regression test-day animal model implemented with the R package “blupADC” [51]. This model incorporated the following fixed effects: herd–test-date (HTD), calving year–season (YS), and month of calving (MC). To account for variations related to the lactation stage and day-to-day fluctuations, fixed regressions involving days in milk (DIM) and third-order Legendre polynomials were used. In addition to these fixed regressions, we also considered individual additive genetic and permanent environmental effects as random regression effects in the model. The model equation used for this analysis is as follows:

$$y_{ijklmn}={HTD}_{i}+{YS}_{j}+{MC}_{k}+\sum_{n=0}^{3}b_{ln}L_{n}\left(w_{t}\right)+\sum_{n=0}^{3}a_{mn}L_{n}\left(w_{t}\right)+\sum_{n=0}^{3}P_{mn}L_{n}\left(w_{t}\right)+e_{ijklmn}$$

where $y_{ijklmn}$ is used to indicate the test-day records (MY, FP, or PP), while ${HTD}_{i}$ denotes the fixed effects of the *i*th herd-test day; ${YS}_{j}$ represents the fixed effects of the *j*th calving year–season and ${MC}_{k}$ specifies the fixed effects of the *k*th month of calving. The $b_{ln}$ are the nth fixed regression coefficients corresponding to the *n*th Legendre polynomials, whereas $a_{mn}$ represent the nth random regression coefficients representing additive genetic effects of the *m*th buffaloes and $P_{mn}$ denote the nth random regression coefficients for permanent environment effects of the *m*th buffaloes. The $L_{n}\left(w_{t}\right)$ are the nth covariate of Legendre polynomials at day *t* in milk (DIMt) and $w_{t}$ signifies the normalized time value at DIMt (DIM = 5, 6, …, 300); and $e_{ijklmn}$ are the random residual effects. The relationship matrix utilized in this model was constructed using data from a relatively small pedigree of 215 animals. This figure is notably modest when compared to the standard practice in dairy cattle studies. The primary reason for this limited number of animals in the pedigree file is the common lack of comprehensive pedigree records in buffalo farming, particularly for animals with complete test-day records during their first three lactations. Notably, previous studies conducted under similar circumstances have provided valuable evidence supporting the effectiveness of using small sample sizes with extreme phenotypes in dairy buffaloes [1,21,52]. This aligns with established practices within buffalo studies for adjusting phenotypes for non-genetic factors and underscores the relevance of our approach.

### 4.2. SNP Genotyping

For the DNA analysis, 5 mL of blood samples were obtained from the previously mentioned 100 individuals. These samples were collected from the jugular vein in sterilized vacutainer tubes coated with EDTA as an anticoagulant. The samples were kept on ice and promptly transferred to the laboratory for further analysis. SNP genotyping was carried out on each of these samples using whole-genome resequencing technology implemented in the Genome Analysis Toolkit v4.2 (GATK) [53] on the Illumina HiSeq 2500 platform. The reference genome employed was the Indian Murrah genome (assembly version: NDDB_SH_1). Subsequently, SNPs were imputed using Beagle version 5.4 software [54]. Following imputation, all imputed SNPs were subjected to quality control procedures utilizing PLINK 1.9 software [55]. The quality control criteria included SNP call rate > 99%, individual call rate > 99%, minor allele frequency (MAF) > 0.95, and Hardy–Weinberg Equilibrium (HWE) with a threshold greater than 1 × 10^−6^. As a result, 100 buffaloes and 679,118 SNPs successfully passed the quality control measures and were used for further analysis.

### 4.3. RNA-Seq and Analyses

For our transcriptome analysis, we collected 10 mL of blood samples from each of the 100 buffaloes with mid-lactation, all drawn from the jugular vein. The samples were immediately preserved in liquid nitrogen before being transferred to −80 °C storage for subsequent RNA extraction. After that, each of the aforementioned 100 samples, yielding a total of 2 μg of RNA per sample, was utilized to construct libraries using the TruSeq RNA Sample Preparation Kit (Illumina, San Diego, CA, USA). Subsequently, we performed sequencing for each library on the Illumina HiSeq 4000 platform (Illumina, San Diego, CA, USA). To ensure data quality, we conducted a quality check for the raw data using fastp ver. 0.23.4 software [56]. The cleaned data were then mapped using the Hisat2 ver2.2.1 software [57], aligning it with the Indian Murrah genome as a reference. Subsequently, the featureCounts function in the Rsubread package [58] was used to create the gene count matrix for the samples studied. Finally, transcripts per million (TPM) values for each gene were obtained using the DESeq2 R package [59], and gene annotation and enrichment analysis were performed with the clusterProfile R package [10].

### 4.4. GWAS Analysis

GWAS for milk production traits in the buffalo population was performed using the Fixed and random model Circulating Probability Unification (FarmCPU) method, implemented in rMVP version 0.99 software [60]. To mitigate potential false positive signals, we adjusted for population structure and relatedness among individuals through principal component analysis (PCA; *n* = 3) and a kinship matrix, respectively. For the purpose of multiple testing corrections, we set the threshold level for Bonferroni correction at *p*-value < 0.05/N, where N represents the number of SNPs that passed quality control criteria (679,118). A significance cutoff of *p*-value < 1 × 10^−4^ and 7.36 × 10^−8^ was selected to indicate nominal and significant associations, respectively.

### 4.5. TWAS Analysis

In this study, a two-stage TWAS approach was employed for the analysis of EBVs for milk production traits in buffaloes using TIGAR-V2 software (https://github.com/yanglab-emory/TIGAR, (accessed on 16 January 2024)) [18]. Initially, we constructed gene expression prediction models using both transcriptomic and genetic data from the same samples. In these models, we used the genotypic data (G) of *cis*-SNPs located within a 1 Mb window surrounding the transcripition start site (TSS) of the target gene (g) as predictors. We assumed an additive genetic model for the expression of quantitative traits ($E_{g}$) in relation to the target gene. The model equation can be represented as follows:

(1) $$E_{g}=G_{w}+\varepsilon ;\;\varepsilon \sim N\left(0,\sigma_{\varepsilon }^{2}I\right)$$

where $E_{g}$ is the gene expression levels for the target gene g, *G* is the genotype matrix encompassing all considered SNP genotypes (encoded as the number of minor alleles of SNPs within a 1 Mb interval of the target gene region), and w is the *cis*-eQTL effect size vector.

To predict the GReX values, TIGAR-V2 employs the estimates of *cis*-eQTL effect sizes and genotype data as inputs. The imputation of GReX values is accomplished using the following formula:

(2) $$\hat{GReX}=G_{new}\hat{w}$$

where $G_{new}$ is the genotype matrix for the new samples. TIGAR-V2 implements nonparametric Bayesian DPR to estimate *w*, which incorporates eQTL effect sizes in a broad sense, irrespective of whether the SNP has a genome-wide significant eQTL *p*-value.

In this analysis, the burden test was utilized to examine the association between GReX values (as a covariate) and the phenotype of interest (as a response variable) based on the general linear regression model. The burden test statistics consist of both FUSION and SPrediXcan Z-score statistics. These statistics were estimated using the following Equations ((3) and (4), respectively):

(3) $$\tilde{Z}g,FUSION=\frac{\sum_{l=1}^{m}\left(\hat{w_{l}}z_{l}\right)}{\sqrt{\hat{w^{’}}V\hat{w}}},\;V=Corr(G_{0})$$

(4) $$\tilde{Z}g,SPrediXcan=\frac{\sum_{l=1}^{m}\left(\hat{w_{l}}\hat{\sigma_{l}}z_{l}\right)}{\sqrt{\hat{w^{’}}V\hat{w}}},\;\hat{\sigma_{l}^{2}}=Var(G_{0,1}),V=Cov(G_{0})$$

where *Z* denotes the Z-score statistic value of genetic variant l by a single-variant GWAS test. The required covariance matrix of linkage disequilibrium (or correlation matrix for the FUSION test statistic) among test *cis*-SNPs (V) and the genotype variance of test *cis*-SNPs can be derived from reference genotype data (G_0_).

### 4.6. Statistical Considerations for TWAS

For the TWAS analysis, we defined the threshold for identifying significant signals as *p*-value < 0.05/N, where N represents the effective number (N = 6389, 6363, and 6228 for MY, FP, and PP, respectively) after Bonferroni correction. We conducted gene annotation of the TWAS signals using Bedtools ver.2.31.0 [61], with the Indian Murrah genome GTF file as the reference. Specifically, we considered a region of approximately 25 kb surrounding the TWAS signals. To explore the protein–protein interaction (PPI) relationship among the TWAS genes, we utilized the STRING database (v12.0).

## 5. Conclusions

In summary, this study harnessed the power of TWAS to identify 227 (55 for MY, 71 for FP, and 101 for PP) potentially causal genes associated with milk production traits, addressing the constraints of prior GWAS for the same traits in buffalo populations. Among these, 9 (*USP7*, *USP16*, *BRCC3*, *EIF3A*, *NAS2*, *RRP8*, *CACUL1*, *DCUN1D4*, and *OCIAD1*), 15 (*SSRP1*, *CSNK2B*, *CBX4*, *KDM5A*, *ACTG1*, *SMAD2*, *SMG1*, *ANKRD32*, *MALT1*, *PUM2*, *LDAH*, *MTO1*, *KPTN*, XP_006074182.1, and XP_006049223.1), and 15 (*RAB7L1*, *NUCKS1*, *PM20D1*, *SLC41A1*, *TMEM161B*, *NCOA7*, *APOBR*, *ATXN2L*, *PRSS53*, XP_006057024.1, *PRPS1*, *TRMT11*, *PUS1*, *ARMCX3*, and *TMEM135*) TWAS genes emerged as promising determinants for MY, FP, and PP traits, respectively. Additionally, our network analysis elegantly pinpointed TWAS genes with potential involvement in mammary gland development and lactation, underscoring their critical roles in milk production performance. While the study boasts strengths like non-parametric Bayesian DPR for *cis*-eQTL weight estimation and comprehensive expression data, it is not immune to limitations, notably the modest sample size and the exclusive focus on blood rather than mammary tissues. Notwithstanding these constraints, our research lays a solid foundation for forthcoming investigations in buffalo genetics and breeding, providing invaluable knowledge for optimizing milk production in this economically vital livestock species.

## Figures and Tables

**Table 1:** GWAS summary for estimated breeding values of milk yield in buffaloes.

SNP	CHR	POS	Effect Size	SE	*p*-Value	Nearest Genes
2_179378512_T_C	2	179378512	−0.343	0.058	4.40 × 10^−11^	*MAN1C1*
3_93238969_G_A	3	93238969	0.164	0.029	1.03 × 10^−9^	*LOC123332809*
4_21904376_A_C	4	21904376	0.287	0.061	1.59 × 10^−8^	*ETV6*
10_18455881_T_C	10	18455881	0.146	0.028	1.81 × 10^−0^	*SASH1*
12_62011074_T_C	12	62011074	−0.248	0.034	7.16 × 10^−14^	*VPS54*
18_422296_C_T	18	422296	−0.215	0.039	4.97 × 10^−10^	*LOC123330224*
19_11892609_A_G	19	11892609	0.562	0.081	8.00 × 10^−14^	*LOC112580602*

Note. GWAS: genome-wide association study, SNP: single nucleotide polymorphism, CHR: chromosome, POS: position, SE: standard error.

**Table 2:** GWAS summary for estimated breeding values of fat percentage in buffaloes.

SNP	CHR	POS	Effect Size	SE	*p*-Value	Nearest Genes
4_86264798_C_T	4	86264798	−0.111	0.019	4.09 × 10^−11^	*SLC38A1*
6_88599507_T_C	6	88599507	0.077	0.013	2.34 × 10^−10^	*DAB1*
12_11038738_T_C	12	11038738	0.102	0.016	1.58 × 10^−11^	*CCT7*
20_56894773_G_T	20	56894773	0.054	0.010	1.19 × 10^−9^	*LOC102392630*

Note. GWAS: genome-wide association study, SNP: single nucleotide polymorphism, CHR: chromosome, POS: position, SE: standard error.

## Data Availability

The data that support the study findings are available from authors upon request.

## Supplementary Materials

The following supporting information can be downloaded at: https://www.mdpi.com/article/10.3390/ijms25052626/s1.
